# Stabilizing Oxygen Redox via Li Dopants Into P2‐Type Cathodes for High‐Voltage Sodium‐Ion Batteries

**DOI:** 10.1002/advs.78593

**Published:** 2026-11-03

**Authors:** Peng Dai, Yanfen Wen, Chenguang Shi, Jie Fu, Jinxia Lin, Hang Li, Sylvio Indris, Jingjing Fan, Yuxiang Xie, Yonglin Tang, Shishi Liu, Chenxu Luo, Yuan Chen, Mohsen Shakouri, Tom Regier, Fang Fu, Jun‐Tao Li, Ling Huang, Shi‐Gang Sun, Ya‐Ping Deng

**Affiliations:** ^1^ State Key Laboratory of Physical Chemistry of Solid Surfaces Collaborative Innovation Center of Chemistry for Energy Materials College of Chemistry and Chemical Engineering Xiamen University Xiamen China; ^2^ Key Laboratory of Functional Materials and Applications of Fujian Province School of Materials Science and Engineering Xiamen University of Technology Xiamen China; ^3^ Institute For Applied Materials Karlsruhe Institute of Technology Eggenstein‐Leopoldshafen Germany; ^4^ Innovation Research Institute in Advanced Electronic Chemicals of Quzhou Quzhou Zhejiang China; ^5^ Canadian Light Source Saskatoon Canada; ^6^ College of Materials Science and Engineering Huaqiao University Xiamen China; ^7^ College of Energy Xiamen University Xiamen China

**Keywords:** anionic redox, lattice oxygen, li doping, P2‐type layered cathode, phase transitions, sodium‐ion batteries

## Abstract

Anionic redox offers a pathway to increase the energy density of layered cathodes for sodium‐ion batteries, yet it is often accompanied by structure and interface degradation. Here, we demonstrate that Li dopants as regulators for oxygen redox in P2‐Na_0.67_Ni_0.33_Mn_0.67_O_2_ through tuning local coordination environments and charge compensation pathways. Li dopants enhance Ni redox activity while moderating transition metal‐oxygen (TM‐O) coupling at high voltages. The online electrochemical mass spectrometry with ^18^O isotope labeling reveals that it suppresses the lattice oxygen participation in electrolyte decomposition and mitigates irreversible oxygen loss. As a result, the Li‐doped Na_0.67_Li_0.2_Ni_0.23_Mn_0.67_O_2_ exhibits improved rate capability cycling stability, delivering an initial capacity of 116.6 mAh/g with 80% retention after 400 cycles at 1 C, compared to 27% for the undoped counterpart. This work highlights the importance of controlling reactive oxygen species and establishes a strategy to stabilize high‐voltage operation by decoupling oxygen redox from degradation pathways in sodium‐ion cathodes.

## Graphical abstract

Regulating anionic redox of layered cathodes provides the promises as performance promotor for sodium ion batteries. In this work, Li doping strategy has been developed to stabilize oxygen redox in the voltage window from 4.1 to 4.3 V. The underlying mechanism has been investigated through integrating a variety of characterizations.

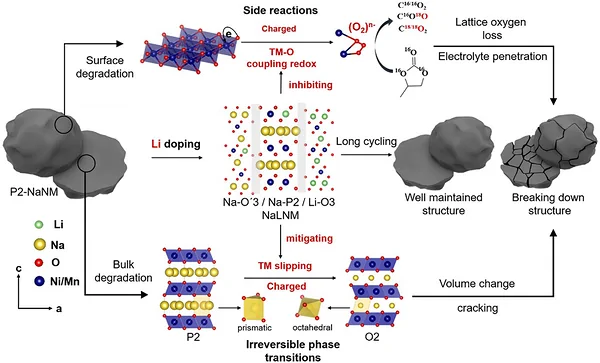

## 1 Introduction

Sodium‐ion batteries (SIBs) have emerged as promising alternatives to lithium‐ion batteries (LIBs) for large‐scale energy storage, owing to the elements abundancy and the wide distribution of sodium resources [1]. Among various cathode candidates, P2‐type layered oxides have attracted considerable attention due to their relatively high specific capacity, elevated operating voltage, environmental benignity, and synthetic compatibility with the established LIBs technologies [2, 3, 4]. In comparison, the intrinsically lower energy density of SIBs remains the critical bottleneck, arising from the higher atomic mass and less favorable redox potential of Na compared to Li [5]. Therefore, substantial efforts have been devoted to simultaneously improving energy density and cycling stability of the P2‐type cathodes [6, 7].

Recent studies have shown that P2‐type layered oxides with strong transition metal‐oxygen (TM‐O) covalency or nonbonding oxygen states induce lattice O redox when exceeding 4.2 V, providing additional capacity beyond the conventional TM redox [8, 9]. Nevertheless, O redox is usually along with the severe structure distortion, gas release, and capacity fading [10, 11]. To address these challenges, various strategies, including heteroatom doping with Li, Mg, Al, and Ti, have been proposed to stabilize the layered structure and suppress detrimental phase transitions, thereby extending cycling lifespan [12, 13, 14, 15]. Despite these advances, the fundamental role of heteroatom dopants in regulating lattice oxygen behavior remains insufficiently understood, particularly regarding their influence on gas evolution, structural integrity, and electrolyte interfacial reactions within the high‐voltage window. Given that O‐related process critically govern interphase instability, elucidating and controlling O redox is essential for developing high‐energy SIB cathodes [16, 17].

In this work, we report a Li‐doping strategy to regulate O redox in P2‐type layered oxides. A Li‐substituted Na_0.67_Li_0.2_Ni_0.23_Mn_0.67_O_2_ has been synthesized as the demonstration, featuring local Li‐O‐□ and Li‐O‐Na coordination environments, where denotes vacancies within Na layer. By combined spatially and temporally resolved characterizations, we show that Li incorporation constrains Na^+^/vacancies rearrangement and suppresses the P2‐O2 phase transition, compared with Na_0.67_Ni_0.33_Mn_0.67_O_2_ (NaNM) prepared via a similar route. Li dopants shift O redox mechanism by enhancing Ni redox activity while moderating O participation in TM‐O coupled redox. This regulation inhibits the formation of reactive (O_2_)^n−^ species and mitigates O‐driven interfacial reactions with the electrolyte, thereby improving structure stability during cycling. As a result, the Li‐doped cathodes enable SIBs with enhanced cyclability and rate capability.

## 2 Results and Discussion

### 2.1 Physiochemical Features of the Pristine Cathodes

As shown in Figure S1, NaNM and NaLNM both display a spherical morphology with a particle diameter of around 15 µm. The inductively coupled plasma‐mass spectrometry (ICP‐MS) results (Table S1) indicate that the two samples with similar stoichiometric TM ratios. The powder x‐ray diffraction (XRD) patterns and corresponding Rietveld refinements are exhibited in Figure 1a,b. NaNM crystallizes in a typical P2‐type layered structure with space group P6_3_/mmc [18]. In contrast, NaLNM exhibits intergrowth phases composed of Na‐P2/Na‐Oˊ3/Li‐O3 domains, where the Na‐Oˊ3 and Li‐O3 phases are assigned to C2/m and R‐3m space groups, respectively (Tables S2, S3) [19]. Meanwhile, high‐resolution transmission electron microscopy (HRTEM) reveals that NaLNM accommodates the intraparticle coexistence of P2‐, Na‐O′3‐, and Li‐O3‐like nanodomains, rather than their existence solely as physically separated secondary particles, as shown in Figure S2.

**FIGURE 1:**
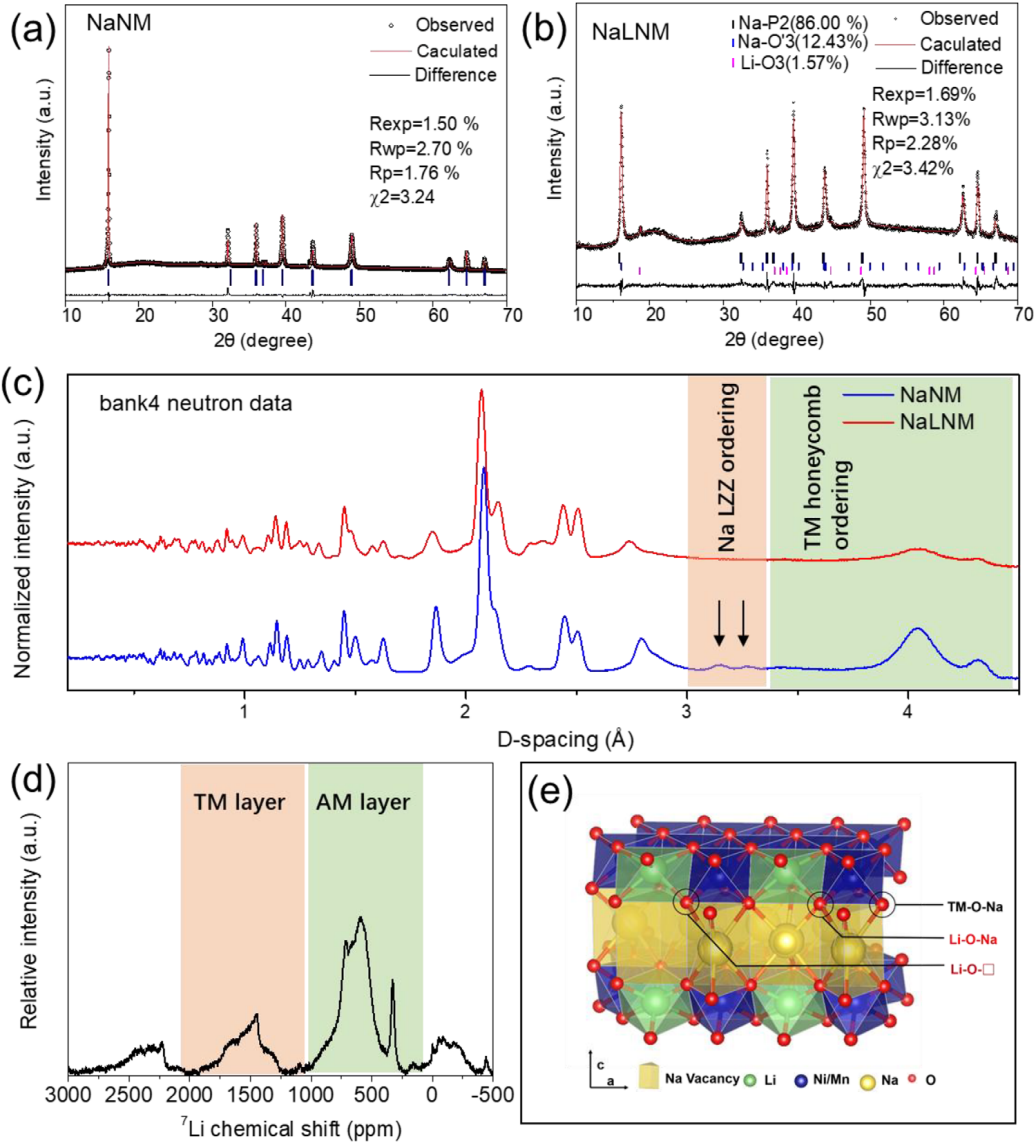
Structural characterization of pristine materials. Powder XRD pattern and corresponding Rietveld refinements of (a) NaNM and (b) NaLNM, (c) NPD profiles highlighting Na‐layer large zigzag (LZZ) ordering and transition metal honeycomb ordering. (d) Ex situ ^7^Li NMR spectrum of pristine NaLNM, indicating Li in both AM layer‐associated and TM layer‐associated local environments (e) Schematic illustration of local oxygen coordination environments in NaLNM, including Li‐O‐□ and Li‐O‐Na configurations.

To investigate cation ordering, neutron powder diffraction (NPD) measurements were performed, taking advantage of the significant difference in neutron scattering lengths between nickel (Ni) and manganese (Mn). As shown in Figure 1c, the reflections in the yellow‐shaded region correspond to large zigzag (LZZ) ordering within the Na layers, while those in the green‐shaded are associated with Ni‐Mn honeycomb ordering within TM layers [20]. Compared with NaNM, NaLNM shows broadened and weakened superlattice reflections, indicating partial disruption of both Na‐layer ordering and TM honeycomb ordering upon Li incorporation. Such structural disorder is expected to suppress Na^+^/vacancy ordering and facilitate Na^+^ diffusion [21].

To gain insights into the local environments of Li, solid‐state nuclear magnetic resonance (ss‐NMR) measurements were conducted on NaLNM. The ^7^Li NMR spectrum (Figure 1d) exhibits two distinct sets of resonances at 250–1000 ppm and 1250–2000 ppm, corresponding to Li^+^ in alkali metal (AM) layer ‐associated and TM layer‐associated, respectively [22]. The integrated intensity is dominated by the alkali‐metal‐layer‐associated region. Therefore, compared to NaNM, Li incorporation introducing distinct local environments, including Li‐O‐□ and Li‐O‐Na configurations, which modify the electronic and structural landscape of oxygen in NaLNM, as demonstrated in Figure 1e.

### 2.2 Electrochemical Behaviors

To electrochemically assess the impact of Li doping, the performance of SIBs employing NaNM and NaLNM cathodes was systematically investigated. Their charging‐discharging profiles are shown in Figure 2a,b. The cells were initially activated at 0.1 C (1 C = 120 mA g^−1^) for three cycles, followed by cycling at 0.2 C. NaNM displays an extended plateau above 4.2 V with approximately 46% capacity; however, this high‐voltage redox is quickly attenuated within 50 cycles. In contrast, NaLNM exhibits smoother voltage profiles, with 75% of the capacity delivered below 4.2 V, and maintains stable electrochemical behavior over prolonged cycling. These results indicate that Li doping effectively improves redox reversibility and suppresses high‐voltage degradation.

**FIGURE 2:**
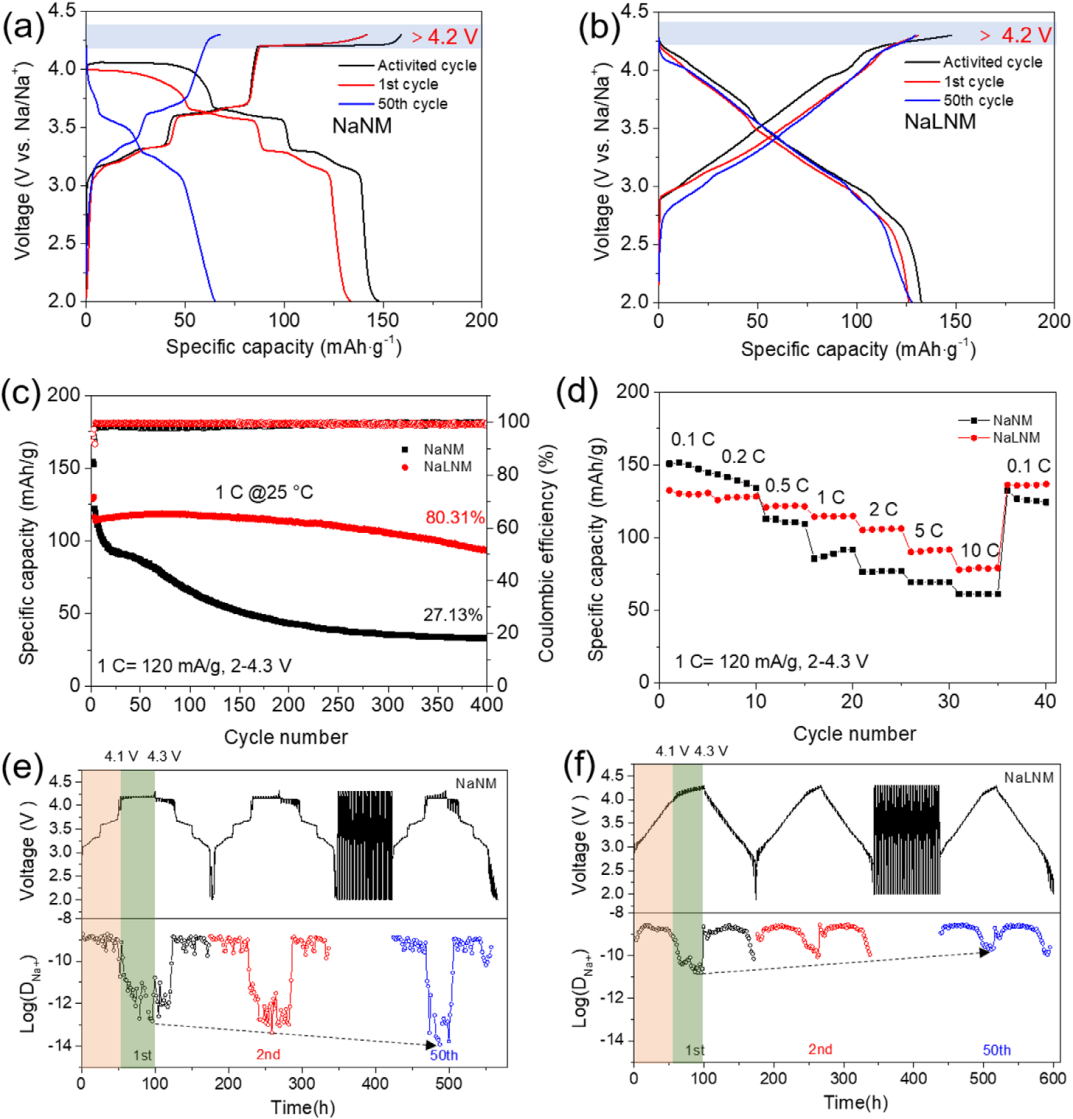
Electrochemical performance of NaNM and NaLNM cathodes. Galvanostatic charge–discharge profiles of (a) NaNM and (b) NaLNM at different cycles. (c) Long‐term cycling performance at 1 C. (d) Rate capability of Na/NaNM and Na/NaLNM cells at 25°C. Na^+^ diffusion coefficients calculated from GITT measurements for (e) NaNM and (f) NaLNM during selected cycles.

Long‐term cycling tests are conducted at 1 C. As shown in Figure 2c, NaLNM exhibits an initial capacity of 116.6 and 93.6 mAh g^−1^ after 400 cycles, with 80.3% capacity retention. In comparison, NaNM exhibits severe capacity decay, retaining only 33.2 mAh g^−1^ with capacity retention of 27.1%. The rate capability of both materials is summarized in Figure 2d. Despite NaLNM exhibiting slightly lower capacities at 0.1 and 0.2 C, it significantly outperforms NaNM at high rates from 0.5 C to 10 C. Specifically, NaLNM delivers capacities of 130.4, 127.7, 121.7, 114.7, 105.9, 91.3, and 78.7 mAh g^−1^ from 0.1 to 10 C, respectively, whereas NaNM maintains only 61.1 mAh g^−1^ at 10 C. When the discharge rate is returned to 0.1 C, NaLNM recovers a capacity of 136 mAh g^−1^, indicating good structural reversibility. This pronounced performance advantage highlights the improved structural stability and accelerated Na^+^ transport kinetics induced by Li incorporation.

To quantitative analysis on Na^+^ transport kinetics, diffusion coefficients (D_Na+_) were calculated by using the galvanostatic intermittent titration technique (GITT). The parameter settings are described in the Experimental Section. Meanwhile, the typical voltage relaxation curves are shown in Figure S3, and the results indicate that the electrode material has approached equilibrium before calculating the Na^+^ diffusion coefficient. For clarity, the charging process is divided into two distinct regions (Figure 2e,f): a low‐voltage region from 2.0 to 4.1 V and a high‐voltage region from 4.1 to 4.3 V. In the low‐voltage region, both materials exhibit comparable D_Na+_ values on the order of 10^−9^ cm^2^ s^−1^, as well as the entire discharging process. However, in the high‐voltage region, NaNM shows a sharp decrease in D_Na+_ to 10^−13^ cm^2^ s^−1^, which further declines to ∼10^−14^ cm^2^ s^−1^ after 50 cycles. For comparison, NaLNM maintains significantly higher diffusion coefficients, and even exhibits a gradual increase upon cycling. These results are consistent with improved electrochemical performance and indicate that Li doping effectively stabilizes the structure and facilitates Na^+^ transport, particularly under high‐voltage conditions.

### 2.3 Structure Evolutions

The structure evolution of NaNM and NaLNM during the sodiation/desodiation processes is investigated through in situ XRD characterization. As shown in Figure 3a, the (002) and (004) peaks of NaNM exhibit a shift toward lower angles during Na^+^ extraction, indicating an expansion along the c‐axis due to increased electrostatic repulsion between adjacent oxygen layers. In the meantime, the (100) and (012) peaks exhibit a shift toward higher angles up to 4.1 V, corresponding to contraction in the *ab* plane, associated with TM oxidation [16]. Above 4.2 V, the (002) peak shows reduced displacement accompanied by a marked decrease in intensity, while a new diffraction feature associated with the O2 phase appears at ∼20° [23]. This indicates the onset of the P2–O2 phase transition. During this process, the Na^+^ coordination transforms from trigonal prismatic to octahedral, resulting in reduced interlayer spacing and impedes Na^+^ diffusion, as supported by D_Na+_ decrease of NaNM in the high‐voltage region (Figure 2e). During the subsequent sodiation process, the (002) peak position exhibits minimal alterations, while its intensity decreases significantly to 56% after the first cycle, suggesting severe structural degradation in the bulk of NaNM. After the second cycle, the (002) peak nearly disappears, indicating severe structure degradation in the bulk of NaNM. In contrast, NaLNM shows highly reversible structure changes during cycling. As shown in Figure 3b, the (002) and (100) peaks shift toward lower and higher angles during Na^+^ extraction process, respectively, suggesting expansion in the *c*‐axis direction and contraction in the *a*‐axis direction. Notably, no O2 phase is detected even when charged to 4.2 V, indicating effective suppression of P2‐O2 phase transition. Moreover, the (002) peak retains 95.4% of its initial intensity after the second cycle, demonstrating significantly improved structural reversibility enabled by Li doping.

**FIGURE 3:**
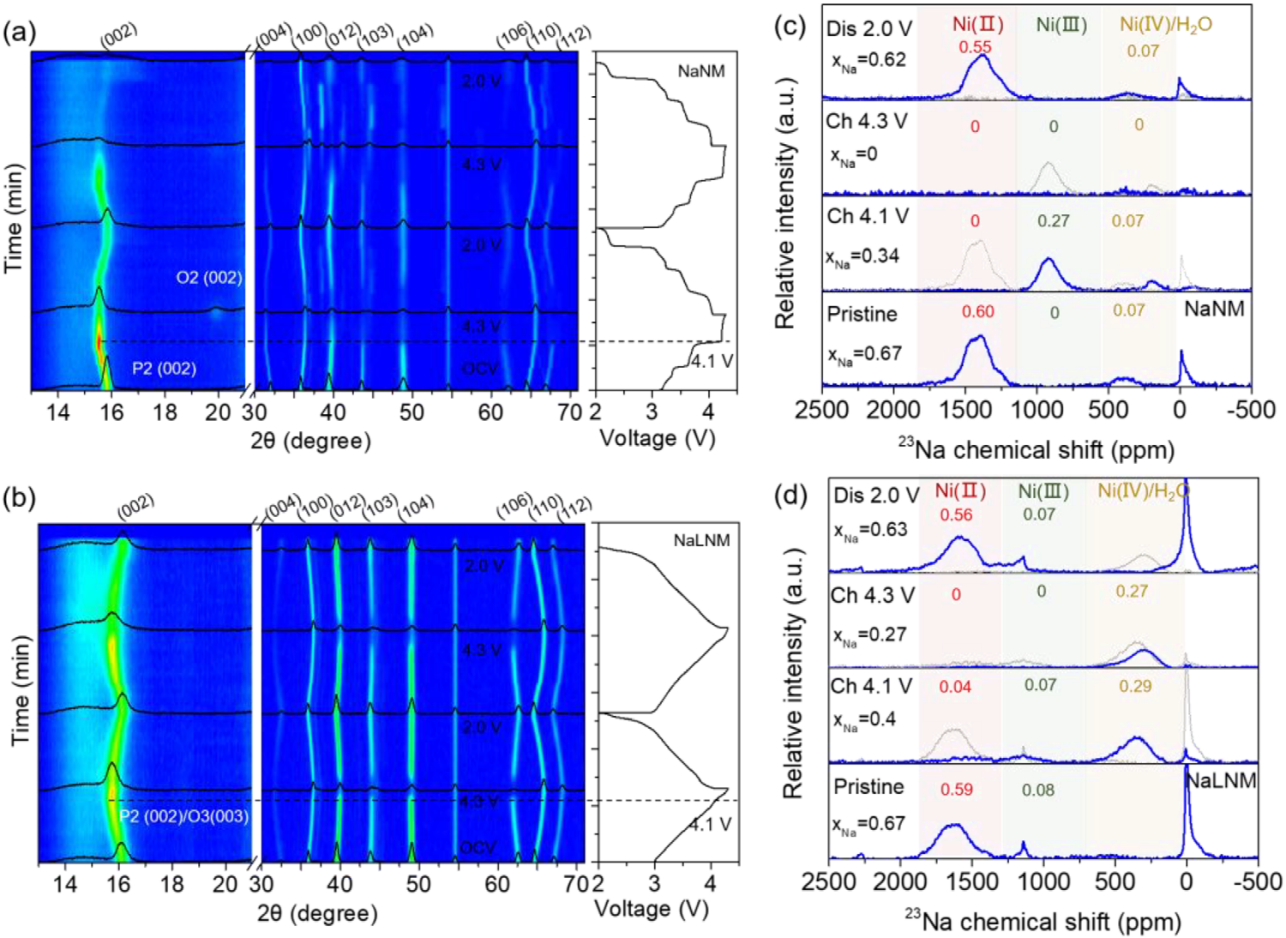
Structural evolution during electrochemical cycling. In situ XRD contour maps and corresponding charge–discharge profiles during initial two cycles of (a) NaNM and (b) NaLNM. Ex‐situ ^23^Na solid‐state NMR spectra of (c) NaNM and (d) NaLNM during the first cycle.

To further gather insights into structural evolution, ex situ ^23^Na ss‐NMR measurements were performed at different electrochemical states (Figure 3c). The pristine NaNM exhibits signals within the ranges of 1250–1750 and 250–500 ppm, which are associated with P2 and hydrated phases, respectively [24]. The peaks at 0 ppm are indicative of phase impurities within diamagnetic environments around Na, resulting from electrolyte decomposition products or residual Na_2_CO_3_ [25]. Upon charging to 4.1 V, the P2 phase‐related peaks experience a chemical shift toward 650–1150 and 0–250 ppm and a decrease in intensity, reflecting Ni oxidation and Na^+^ extraction. When further charging to 4.3 V, the absence of all ^23^Na resonance indicates nearly complete Na^+^ extraction, consistent with the formation of the O2 phase. Upon discharging to 2 V, the P2 phase‐related peak intensity peak recovers only partially, reflecting incomplete Na^+^ reinsertion and irreversible structural degradation. For comparison, ^23^Na NMR spectra of NaLNM (Figure 3d) showcase two dominant signals peaks at 1300–1900 and 1000–1250 ppm, corresponding to Na‐P2 and Na‐Oˊ3 phases, respectively [19]. The substitution of Ni^2+^ by Li^+^ in the TM layer increases the average isotropic shift of Na in the P2 phase. Meanwhile, the lack of hydrated phase signals in NaLNM suggests that Li doping suppresses water incorporation. During charging to 4.1 V, a new resonance appears at 250–750 ppm, with an intensity of 0.29. Upon further charging to 4.3 V, the signal decreases slightly to 0.27, along with gradual weaken but remain detectable of Na‐P2 and Na‐O'3 signals. This indicates incomplete Na^+^ extraction, with residual Na^+^ stabilizing the TM layers and inhibiting P2‐O2 phase transition. Upon discharging back to 2 V, resonance signals at 250–750 ppm disappears due to Ni reduction, while the peak intensity of the Na‐P2 phase decreased by 5.1% relative to the pristine state, which is less than the 8.3% decrease observed for NaNM.

### 2.4 Charge Compensation

The in situ Raman spectroscopy was conducted to monitor changes of TM‐O coordination during the initial charging process at 0.2 C. As presented in Figure 4a,b, the pristine NaNM exhibits two prominent peaks at 486 and 593 cm^−1^, corresponding to the E_g_ and A_1g_ vibrational modes of TM‐O bonds (Figure S4) [26]. Upon charging, their intensities gradually decrease, alongside with the emergence of a new peak at 524 cm^−1^, which reaches maximum intensity at 4.1 V. The initial decline of A_1g_ and E_g_ vibrations is ascribed to Ni oxidation from Ni^2+^ to Ni^3+^, associated with Jahn–Teller distortion [27]. Further charging to 4.1 V leads to oxidation of Ni^3+^ to Ni^4+^, accompanied by the disappearance of Jahn‐Teller distortion at Ni‐O octahedral and the appearance of the Ni^4+^‐O vibrational mode at 524 cm^−1^ [28]. Upon charging to 4.3 V, Ni^4+^‐O vibrational of NaNM experiences 38% intensity decrease, owing to lattice oxygen loss and the formation of oxygen vacancies [28]. Meanwhile, a new peak at 537 cm^−1^ appears, which is attributed to Ni^4+^‐O vibrations in the O2 phase, confirming the occurrence of the P2‐O2 phase transition. In contrast, NaLNM (Figure 4c,d) exhibits similar spectral evolution but with significantly reduced intensity decay. The extra Ni^4+^‐O vibration peak at 539 cm^−1^ shows ∼28% less attenuation between 4.1 and 4.3 V compared to NaNM, indicating suppressed lattice oxygen loss upon Li incorporation.

**FIGURE 4:**
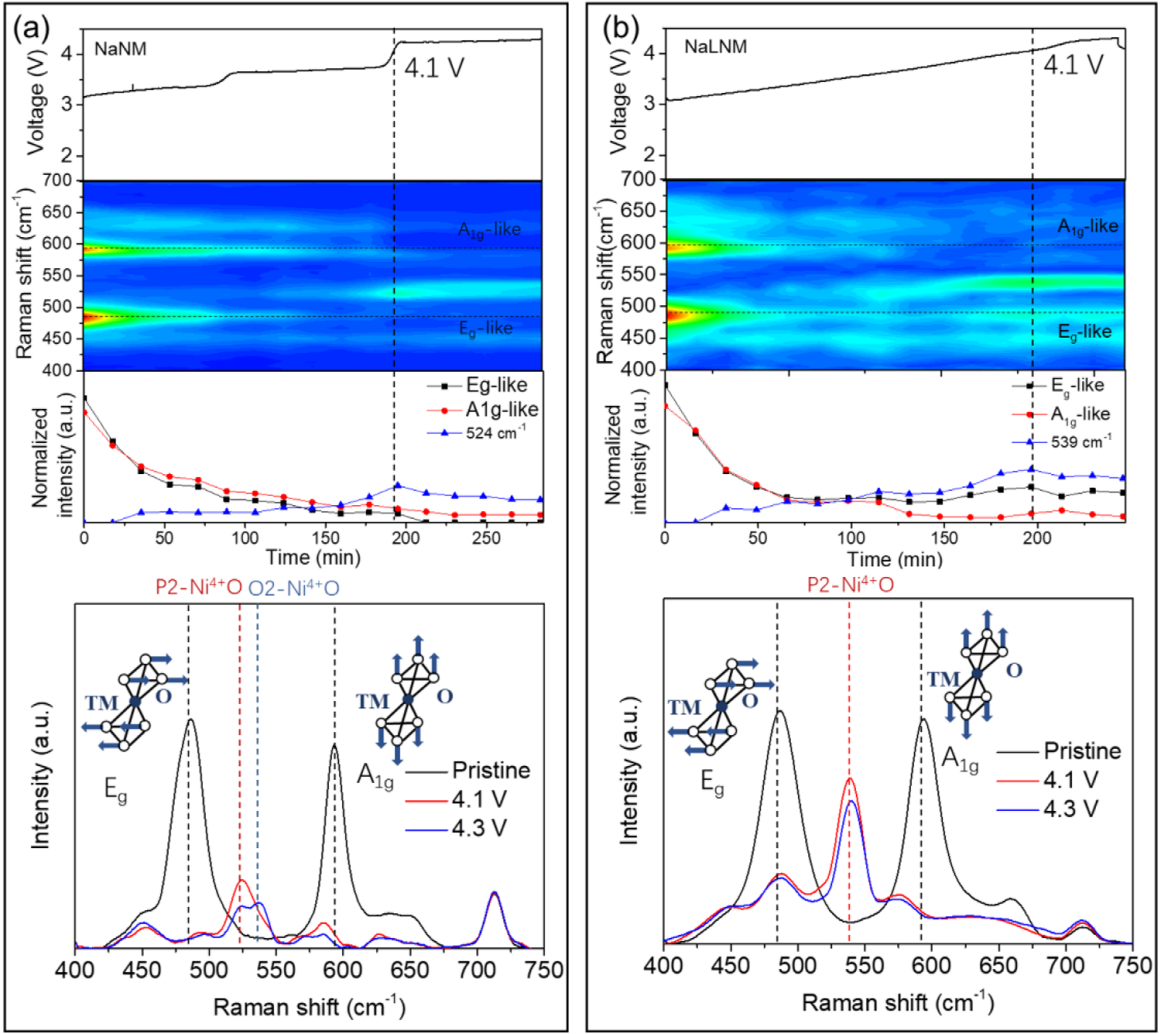
In situ Raman analysis of charge compensation behavior. (a) In situ Raman contour map and (b) Representative Raman spectra at selected voltages, highlighting the evolution of TM‐O vibrations and the emergence of Ni^4+^‐O feature for NaNM; (c) contour map and (d) representative Raman spectra for NaLNM.

To further analyze the roles of Mn and Ni in charge compensation, ex situ X‐ray absorption near‐edge structure (XANES) were employed. Mn *K*‐edge spectra (Figure S5) remain essentially unchanged during cycling for both materials, indicating that Mn is largely electrochemically inactive. In contrast, Ni K‐edge XANES spectra (Figure 5a,b) reveal distinct behaviors. For NaNM in Figure 5a, the absorption edge shifts to higher energy upon charging to 4.1 V, reflecting Ni oxidation. However, further charging to 4.3 V induces a reverse shift toward lower energy, indicating partial reduction of Ni associated with the activation of oxygen redox. This behavior suggests a coupled TM‐O redox mechanism in NaNM, where electrons transfer from O^2−^ to the adjacent TM ions results in oxygen oxidation and formation of intermediate (O_2_)^n−^ species [29, 30, 31]. When discharging back to 2 V, the Ni K‐edge position remains lower than that of the pristine state, indicating irreversible oxygen loss [32].

**FIGURE 5:**
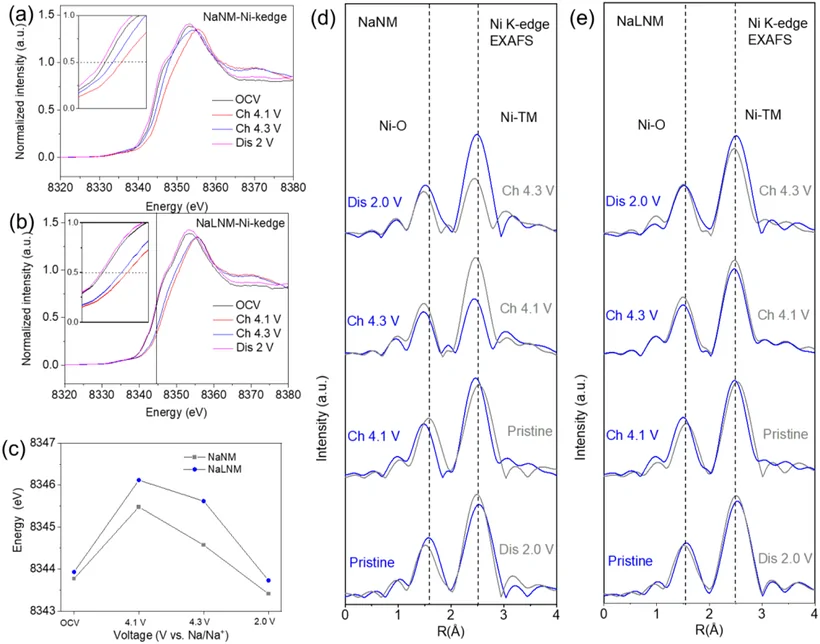
Ni K‐edge XAS analysis of charge compensation behavior. Ni K‐edge XANES spectra of (a) NaNM, (b) NaLNM at different electrochemical states. (c) Evolution of the absorption edge energy E_0.5_ for NaNM and NaLNM. Fourier transform of EXAFS spectra (R‐space) of Ni K‐edge for (d) NaNM and (e) NaLNM, illustrating changes in the local coordination environment during cycling. Grey curves are included as references to highlight spectral evolution of the adjacent curves.

Comparatively, NaLNM exhibits a slight negative shift in the Ni K‐edge upon charging from 4.1 to 4.3 V and upon subsequent discharging to 2 V (Figure 5b). Quantitative analysis of the absorption edge energy (E_0.5_, defined at normalized intensity of 0.5) further supports this trend (Figure 5c) [33]. When charged to 4.1 V, NaLNM shows a larger increase in E_0.5_ compared to NaNM increased (1.7 eV for NaNM and 2.2 eV for NaLNM), indicating more effective Ni oxidation. Notably, the facilitation of Ni oxidation could further mitigate the lattice oxygen oxidation at high voltage. Additionally, the decrease in E_0.5_ from 4.1 to 4.3 V is significantly smaller in NaLNM (0.9 eV for NaNM and 0.5 eV for NaLNM), suggesting less Ni reduction. Together with the O K‐edge sXAS and ^18^O‐OEMS results, these changes support oxygen‐related TM─O charge compensation above 4.1 V. After discharging to 2 V, NaLNM exhibits a smaller decrease in E_0.5_ compared to its OCV state, further indicating improved Ni redox reversibility.

The local coordination environment of Ni was further probed by Fourier‐transformed Ni *K*‐edge extended X‐ray absorption fine spectra (EXAFS) (Figure 5d,e). For NaNM, two main peaks at ∼1.5 and ∼2.6 Å correspond to interatomic Ni‐O and Ni‐TM scattering paths, as shown in Figure 5d [34]. When charging to 4.1 V, the Ni‐O peak shifts to a shorter distance with reduced intensity, reflecting increased oxidation state and decreased coordination number associated with Jahn‐Teller distortion of Ni^3+^ ions [35]. At 4.3 V, further attenuation of the Ni‐O peak indicates reduced coordination, consistent with the oxygen loss phenomenon. After discharging to 2 V, the Ni‐O peak intensity does not fully recover, indicating irreversible structural degradation. In contrast, NaLNM shows a shift of the Ni‐O peak toward shorter distance together with increased intensity at 4.1 V (Figure 5e). In combination with the Ni K‐edge EXAFS results, this behavior indicates Li doping raises the average Ni oxidation state in NaLNM. Accordingly, some Ni already exists as Ni^3+^ in the pristine state, and is further oxidized to Ni^4+^ during charging, consistent with the enhanced Ni‐O scattering intensity. When further charging to 4.3 V, the Ni‐O peak shows only a slight shift to longer distance together with modest intensity attenuation, suggesting limited Ni^4+^reduction toward Ni^3+^, accompanied by partial Jahn‐Teller distortion and minor lattice oxygen loss [26]. Besides, Ni‐O peak intensity at 2 V is largely recovered, indicating the reversible evolution of the coordination structure. These results suggest that Li doping mitigates irreversible lattice oxygen loss and improves structural reversibility at high voltages.

### 2.5 Oxygen‐Related Electronic Changes and Interfacial Side Reactions

To probe charge‐induced changes in oxygen‐related electronic states, O K‐edge soft X‐ray absorption spectroscopy (sXAS) were collected for the pristine and charged (4.3 V) states. As shown in Figure S6, both NaNM and NaLNM exhibit two main features at 530.6 and 533 eV, respectively. These peaks are attributed to hybridization between unoccupied O 2*p* and TM 3*d* orbitals [36]. At 4.3 V, NaNM and NaLNM both present peaks at 532 eV due to oxygen participation in high‐voltage charge compensation [37, 38].

To further elucidate lattice oxygen participation, isotopically labeled ^18^O was introduced as lattice oxygen within NaNM and NaLNM. Time‐of‐flight secondary ion mass spectrometry (TOF‐SIMS) mapping (Figure S7 and our previous work [39]) confirms uniform ^18^O distribution throughout the particle, ensuring reliable tracing of lattice oxygen during electrochemical reactions.

The ^18^O‐labeled samples were subsequently investigated using online electrochemical mass spectrometry (OEMS) measurements (Figure 6). During the initial charging process, no direct O_2_ evolution is detected for either material, while CO_2_ gas is detected as evidence for electrolyte oxidation. Importantly, the presence of ^18^O‐labeled CO_2_ identifies the involvement of lattice oxygen in electrolyte oxidation. In the case of NaNM (Figure 6a), the generation of unlabeled CO_2_ (C^16/16^O_2_) initiates at 3.35 V, which originates from self‐decomposition of PC solvent. When reaching 4.1 V, the appearance of C^16/18^O_2_ and C^18/18^O_2_ signals suggests the onset of lattice oxygen loss. The formation of C^18/18^O_2_ is from the reactions between reactive lattice‐derived oxygen (O_2_)^n−^ species and the electrolyte [40]. With increasing charging voltage, the CO_2_ evolution rate rises significantly, reaching maximum values of 31.36, 13.04, and 4.12 nmol s^−1^ g^−1^ for C^16/16^O_2_, C^16/18^O_2_, and C^18/18^O_2_, respectively at ∼4.2 V. Notably, the CO_2_ evolution profile exhibits a double‐peak shape between 4.1 and 4.3 V, suggesting dynamic competition between consumption of reactive oxygen species and continued electrolyte decomposition. In contrast, NaLNM (Figure 6b) exhibits significantly suppressed gas evolution. The C^16^/^16^O_2_ signal appears at 3.35 V but reaches a much lower maximum rate of 1.84 nmol s^−1^ g^−1^ at 4.26 V. The labeled species C^16/18^O_2_ and C^18/18^O_2_ emerge at 3.66 V, but remain at substantially lower rates. At 4.3 V, their maximum evolution rates are only 2.88 and 1.84 nmol s^−1^ g^−1^, respectively. Comparing with NaNM (Figure S8), the total release of C^16/16^O_2_, C^16/18^O_2_ and C^18/18^O_2_ in NaLNM is reduced by 73.26%, 85.25% and 71.80%, respectively, demonstrating that Li doping effectively suppresses lattice oxygen loss in parasitic reactions.

**FIGURE 6:**
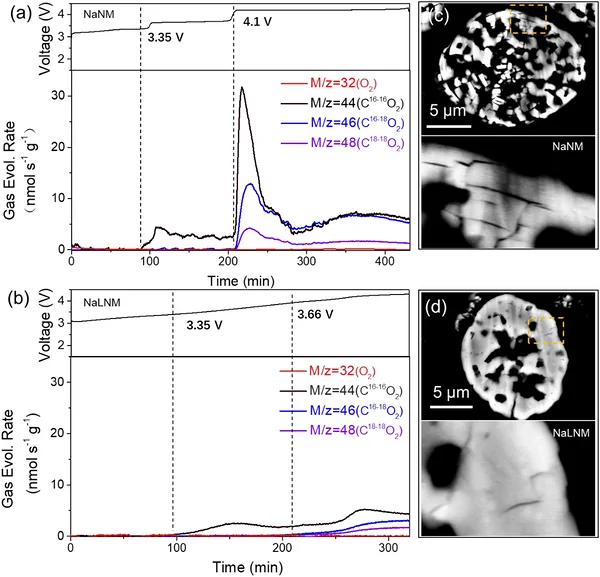
Operando gas analysis and morphological evolution. OEMS profiles during the initial charging process at 0.2 C for ^18^O isotope labelled (a) NaNM and (b) NaLNM, showing the evolution of CO_2_ species (m/z = 44, 46, and 48) associated with electrolyte decomposition and lattice oxygen participation. Cross‐sectional SEM images of (c) NaNM and (d) NaLNM after 100 cycles at 1 C, highlighting structural degradation and crack formation.

The above results indicate Li incorporation regulates reactive oxygen species and mitigates interfacial side reactions between lattice oxygen and the electrolyte. In NaNM, extensive lattice oxygen loss triggers irreversible phase transitions and crack formation (Figure 6c and Figure S9a–d). In contrast, NaLNM suppresses electrolyte decomposition and oxygen release, thereby preserving structural stability (Figure 6d and Figure S9e–h). Further evidence is provided by Raman spectra (Figure S10), where post‐cycling NaNM exhibits a characteristic peak at 634 cm^−1^ assigned to A_1g_ mode of a spinel phase, confirming irreversible phase transition [41]. This feature is absent in the post‐cycling NaLNM, indicating effective suppression of surface degradation and phase transition.

## 3 Discussion

Based on the above structure and spectroscopic characterizations, the degradation of NaNM originates from coupled instabilities in both the bulk and interfacial domains. As illustrated in Figure 7, nearly complete Na^+^ extraction at 4.3 V induces an irreversible P2‐O2 phase transition in the bulk, leading to the coexistence of P2 and O2 phases. This phase heterogeneity generates substantial internal stress and results in the formation of cracks inside particles. Simultaneously, TM‐O coupled processes activated redox above 4.1 V d are consistent with oxygen‐related TM─O charge redistribution and the formation of reactive lattice‐derived oxygen species. These reactive species accumulate near the surface and aggravate electrolyte decomposition, as evidenced by the evolution of isotopically labeled CO_2_ (C^16/18^O_2_ and C^18/18^O_2_). The combined effects of bulk phase transitions and interfacial parasitic reactions lead to continuous lattice oxygen loss and rapid structural degradation in NaNM. In contrast, Li‐doped NaLNM with a hybrid Na‐P2/Na‐Oˊ3/Li‐O3 structure exhibits significantly improved structural stability. Partial retention of Na^+^ at high voltage helps stabilize the TM layers and effectively suppresses P2‐O2 phase transition. Moreover, Li incorporation enhances the reversibility of Ni redox and moderates TM‐O coupled redox processes. As a result, the formation of reactive oxygen species is substantially reduced, leading to suppressed interfacial side reactions and mitigated lattice oxygen loss.

**FIGURE 7:**
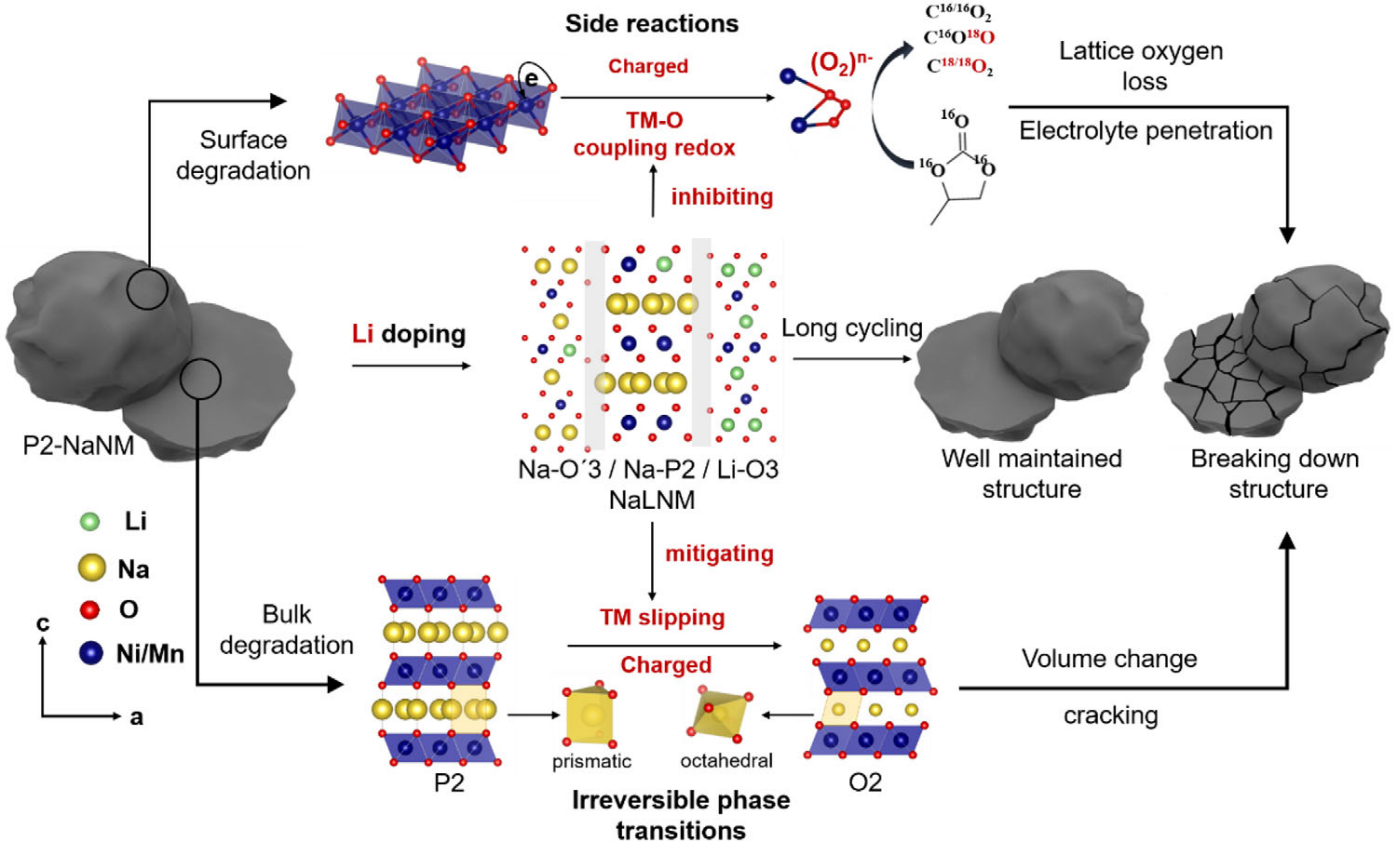
Schematic illustration of the Li‐doping mechanism in P2‐type layered cathodes.

## 4 Conclusion

In this work, Li doping is demonstrated to fundamentally regulate the interplay between bulk structure evolution and interfacial oxygen chemistry in P2‐type layered cathodes. By stabilizing Na occupancy and modifying local coordination environments, Li incorporation suppresses irreversible phase transitions while maintaining structural coherence during high‐voltage operation. More importantly, Li doping alters the charge compensation pathway by promoting TM redox and limiting excessive oxygen participation, thereby reducing the formation of reactive oxygen species and mitigating parasitic interfacial reactions. This dual regulation of bulk and surface processes effectively suppresses lattice oxygen loss and enables sustained electrochemical stability. These findings highlight that controlling reactive oxygen species and their interfacial reactivity is critical for achieving durable high‐voltage performance, and provide a practical strategy for designing advanced sodium‐ion cathodes through targeted modulation of anionic redox behavior.

## 5 Experimental Section/Methods

### 5.1 Material Synthesis and Electrolytes Preparation

Ni_0.33_Mn_0.67_CO_3_ and Ni_0.23_Mn_0.67_CO_3_ precursors were synthesized using a co‐precipitation method. A 2 M aqueous solution of MnSO_4_ and NiSO_4_ was continuously pumped into a stirred tank reactor at a flow rate of 0.6 mL min^−1^, along with separate solutions of Na_2_CO_3_ and NaHCO_3_. The reaction was carried out under controlled conditions: temperature at 50°C, stirring speed at 550 rpm, and pH maintained at 8.0. After 20 h of stirring under an argon atmosphere, the resulting precipitate was thoroughly washed with deionized water and ethanol, and then dried overnight at 90°C in a vacuum oven. Subsequently, each precursor was uniformly mixed with stoichiometric amounts of Na_2_CO_3_ and Li_2_CO_3_, preheated at 500°C for 5 h, and finally annealed at 900°C for 24 h in a muffle furnace to obtain Na_0.67_Ni_0.33_Mn_0.67_O_2_ (NaNM) and Na_0.67_Li_0.2_Ni_0.23_Mn_0.67_O_2_ (NaLNM), respectively. The as‐synthesized NaNM and NaLNM powders were stored in vacuum‐sealed containers. To introduce ^18^O isotope labeling on the surface of the materials, pristine samples were heated in a tube furnace under ^18^O_2_ atmosphere at 600°C for 6 h.

### 5.2 Electrochemical Measurements

The active materials, acetylene black, and polyvinylidene fluoride (PVDF) were homogeneously mixed in a weight ratio of 7:2:1 in N‐methyl‐2‐pyrrolidinone (NMP). The resulting slurry was coated onto aluminum foil and dried at 110°C for 12 h. The dried electrode was punched into circular discs with a diameter of 12 mm and an active mass loading of approximately 2.5 mg cm^−2^. Coin cells of the 2025‐type were assembled in an argon‐filled glovebox with H_2_O and O_2_ levels maintained below 0.1 ppm, using sodium metal as the anode and glass fiber as the separator. The electrolyte consisted of 1.0 M NaClO_4_ in propylene carbonate (PC) containing 5 wt.% fluoroethylene carbonate (FEC). The assembled cells were subjected to galvanostatic charge–discharge cycling at 0.1 C for the first three cycles, followed by cycling at 0.2 C and 1 C for subsequent cycles, within a voltage window of 2.0–4.3 V using a Neware battery tester (CHN) at 25°C. The GITT measurements were performed under the following conditions: Charge current: 0.1 C (1 C = 120 mA g^−1^), Current pulse duration: 15 min (900 s), Relaxation time between pulses: 3 h (10,800 s), Voltage window: 2.0–4.3 V (vs. Na^+^/Na), Temperature: 25°C. The chemical diffusion coefficient of Na^+^ (D_Na_^+^) was calculated using the classical GITT equation derived by Weppner and Huggins [42]:

$$\;D=\frac{4}{\pi \tau }\;{\left(\frac{n_{m}V_{m}}{A}\right)}^{2}{\left[\frac{\left(\Delta E_{s}\right)}{\left(\Delta E_{\tau }\right)}\right]}^{2}$$

where:τ = pulse duration (900 s), n_m_ = molar amount of active material (calculated from electrode mass and molar mass), V_m_ = molar volume of the active material (obtained from crystallographic data), A = geometric electrode area (or electrochemically active surface area, as specified), ΔE_s_ = change in the steady state voltage between two consecutive relaxation steps (i.e., ΔE_si+1_ = Es_i+1_−Es_i_), ΔE_τ_ = voltage change during the current pulse, corrected for the instantaneous IR drop.

### 5.3 Material Characterization

The morphologies of pristine NaNM and NaLNM were characterized using scanning electron microscopy (SEM, Hitachi S‐4800). X‐ray diffraction (XRD) patterns were collected on a Bruker D8 diffractometer (Germany) employing Cu Kα radiation. Rietveld refinements of the XRD data were performed using TOPAS 4.2 software. Raman spectra were acquired on a Renishaw spectrometer (UK) with a 632.75 nm laser excitation source, utilizing a home‐built in situ Raman cell. Time‐of‐flight secondary ion mass spectrometry (TOF‐SIMS) measurements were conducted on a PHI nanoTOF II instrument (Japan) with a 30 keV Bi_3_^+^ primary ion beam. Mn and Ni K‐edges X‐ray absorption spectroscopy (XAS) measurements were carried out at multiple beamlines, including 1W1B beamline at Beijing Synchrotron Radiation Facility (BSRF), Tender X‐ray Beamline at High‐Energy Photon Source (HEPS), Soft X‐ray Microcharacterization Beamline (SXRMB), and BioXAS‐Spectroscopy beamlines at Canadian Light Source (CLS), while O K‐edge data were collected at the Spherical Grating Monochromator (SGM) Beamline of CLS. All XAS data were analyzed using Athena and Artemis software. Online electrochemical mass spectrometry (OEMS) experiments were performed in a custom‐designed OEMS cell coupled to a mass spectrometer (modified Agilent 5975C mass selective detector). Helium was used as the carrier gas at a flow rate of 5 mL min^−^^1^. Local Na and Li coordination environments were investigated by magic‐angle spinning (MAS) ^7^Li and ^23^Na nuclear magnetic resonance (NMR) spectroscopy using a Bruker Advance Neo Spectrometer (Germany) operating at a magnetic field of 4.7 T, corresponding to resonance frequencies of 77.8 MHz (^7^Li) and 52.9 MHz (^23^Na), with samples spun in 1.3 mm rotors at 60 kHz. Neutron powder diffraction (NPD) measurements were conducted in time‐of‐flight (TOF) mode on the Multiphysical Spectrometer (MPI) at the Spallation Neutron Source in China, using a sample mass of 1.5 g.

## Author Contributions

**Peng Dai**: conceptualization, methodology, data curation, formal analysis, writing – original draft. **Yanfen Wen**: data curation, formal analysis, writing – original draft, methodology. **Chenguang Shi**: formal analysis, data curation, investigation, writing – original draft, methodology. **Jie Fu**: data curation. **Jinxia Lin**: data curation, investigation. **Hang Li**: data curation. **Sylvio Indris**: data curation. **Jingjing Fan**: formal analysis. **Yuxiang Xie**: formal analysis, investigation. **Yonglin Tang**: data curation, investigation. **Shishi Liu**: data curation, formal analysis. **Chenxu Luo**: formal analysis, investigation. **Yuan Chen**: data curation, formal analysis. **Mohsen Shakouri**: data curation. **Tom Regier**: data curation. **Fang Fu**: investigation, formal analysis. **Jun‐Tao Li**: validation, data curation. **Ling Huang**: supervision, funding acquisition, resources, writing – review and editing, project administration, conceptualization. **Shi‐Gang Sun**: supervision, resources, writing – review and editing, funding acquisition, project administration, conceptualization. **Ya‐Ping Deng**: funding acquisition, resources, supervision, project administration, writing – review and editing, data curation, conceptualization.

## Conflicts of Interest

The authors declare no conflicts of interest.

## Supporting information




**Supporting File**: advs78593‐sup‐0001‐SuppMat.docx.

## Data Availability

The data that support the findings of this study are available from the corresponding author upon reasonable request.
