# Supplementary material for: Stabilizing Oxygen Redox via Li Dopants Into P2‐Type Cathodes for High‐Voltage Sodium‐Ion Batteries
Source: Adv Sci (Weinh). 2026 Nov 3:e78593. Online ahead of print. doi: 10.1002/advs.78593 (PMC13731074; doi:10.1002/advs.78593)
Supplement: Supplementary file 1 [file ADVS-9999-e78593-s001.docx]

**Stabilizing Oxygen Redox via Li dopants into P2-type Cathodes for High-Voltage Sodium-Ion Batteries**

Peng Dai ^1, #^, Yanfen Wen ^3, #^, Chenguang Shi ^2, 7, #,^ *, Jie Fu ^1^, Jinxia Lin ^1^, Hang Li ^2^, Sylvio Indris ^2^, Jingjing Fan ^1^, Yuxiang Xie ^1^, Yonglin Tang ^1^, Shishi Liu ^1^, Chenxu Luo ^1^, Yuan Chen ^1^, Mohsen Shakouri^4^, Tom Regier^4^, Fang Fu^5^, Jun-Tao Li^6^, Ling Huang ^1, 7^, Shi-Gang Sun ^1, 7,^ *, Ya-Ping Deng ^1,^ *

^1^State Key Laboratory of Physical Chemistry of Solid Surfaces, Collaborative Innovation Center of Chemistry for Energy Materials, College of Chemistry and Chemical Engineering, Xiamen University, Xiamen 361005, China

^2^Institute for Applied Materials, Karlsruhe Institute of Technology, Eggenstein-Leopoldshafen, 76344, Germany

^3^Key Laboratory of Functional Materials and Applications of Fujian Province, School of Materials Science and Engineering, Xiamen University of Technology, Xiamen 361024, China

^4^Canadian Light Source, Saskatoon, SK S7N 2V3, Canada

^5^College of Materials Science and Engineering, Huaqiao University, Xiamen, 361021, China

^6^College of Energy, Xiamen University, Xiamen 361005, China.

^7^Innovation Research Institute in Advanced Electronic Chemicals of Quzhou, QuZhou, Zhejiang 324000, China;

^#^These authors contributed equally: Peng Dai, Yanfen Wen, Chenguang Shi

*E-mail: cgshi@stu.xmu.edu.cn (C.-G. Shi); sgsun@xmu.edu.cn (S.-G. Sun); dyp@xmu.edu.cn (Y.-P. Deng)

**Figure S1** SEM images of pristine (a) NaNM and (b) NaLNM.

**Table S1** ICP-MS results of NaNM and NaLNM

| Materials | Na | Li | Ni | Mn |
| --- | --- | --- | --- | --- |
| NaNM | 0.6835 | 0 | 0.3427 | 0.6713 |
| NaLNM | 0.6813 | 0.2484 | 0.2668 | 0.6694 |

**Table S2** Rietveld refinement results of NaNM; the constrained parameters have been underlined.

| Materials | Atom | X | y | z | Occupancy | Biso |
| --- | --- | --- | --- | --- | --- | --- |
| NaNM | Ni | 0 | 0 | 0 | 0.3345 | 1.226 |
|  | Mn | 0 | 0 | 0 | 0.6655 | 1.226 |
|  | O | 2/3 | 1/3 | 0.08844 | 1 | 0.9259 |
|  | Na1 | 0 | 0 | 1/4 | 0.264 | 1.424 |
|  | Na2 | 1/3 | 2/3 | 1/4 | 0.406 | 1.834 |
| Cell parameters | a=2.883(9)Å | c=11.147(0)Å | V=80.291(6)Å^3^ |  |  |  |
| Rexp=1.50% | Rwp=2.70% | Rp=1.76% | χ^2^=3.24 |  |  |  |

**Table S3** The Rietveld refinement results of NaLNM; the constrained parameters have been underlined.

| Materials | Atom | x | y | z | Occupancy | Biso |
| --- | --- | --- | --- | --- | --- | --- |
| Na-P2 | Li | 0 | 0 | 0 | 0.1 | 0.1776 |
|  | Ni | 0 | 0 | 0 | 0.2411 | 0.1776 |
|  | Mn | 0 | 0 | 0 | 0.6589 | 0.1776 |
|  | O | 1/3 | 2/3 | 0.08427 | 1 | 0.8513 |
|  | Na1 | 0 | 0 | 1/4 | 0.4278 | 0.8692 |
|  | Na2 | 2/3 | 1/3 | 1/4 | 0.2422 | 1.85 |
| Cell parameters | a=2.888(8)Å | c=11.080(7)Å | V=80.085(5) Å^3^ |  |  |  |
| Na-Oˊ3 | Na | 0 | 1/2 | 1/2 | 0.7391 | 0.1563 |
|  | Ni | 0 | 0 | 0 | 0.2548 | 0.9308 |
|  | O | 0.27982 | 0 | 0.77302 | 1 | 0.9333 |
|  | Mn | 0 | 0 | 0 | 0.6452 | 0.9308 |
| Cell parameters | a=5.381(7)Å | b=2.641(1)Å | c=5.742(3) Å | V=78.123(1) Å^3^ |  |  |
| Li-O3 | Li1 | 0 | 0 | 0 | 1 | 0.3041 |
|  | Ni1 | 0 | 0 | 1/2 | 0.3291 | 0.3153 |
|  | Mn | 0 | 0 | 1/2 | 0.6709 | 0.3153 |
|  | O | 0 | 0 | 0.2411 | 1 | 0.5849 |
| Cell parameters | a=2.840(7)Å | c=14.282(1)Å | V=99.814 (2) Å^3^ |  |  |  |
| Rexp=1.69% | Rwp=3.13% | Rp=2.28% | χ^2^=3.42 |  |  |  |

**Figure S2.** The HRTEM image and local FFT analysis of NaLNM. The cyan, green, and orange dashed boxes indicate spatially separated regions within the same primary particle that are consistent with P2-, Li–O3-, and Na–O′3-like nanodomains, respectively. The FFT pattern of the P2-like region shows reflections with lattice spacings of approximately 0.54 and 0.27 nm, corresponding to the (002) and (004) planes, respectively. The Li–O3-like region exhibits approximately collinear reflections with spacings of approximately 0.48 and 0.24 nm, which are assigned to the (003) and (006) planes, respectively. For the Na–O′3-like region, the measured spacings of approximately 0.54 and 0.26 nm are assigned to the (001) and (200) planes. The angle between these two reflections is approximately 72°, consistent with the calculated reciprocal-lattice angle of ~72° for the monoclinic C2/m structure viewed along the [010] zone axis. The phase assignments were made by comparison with the lattice parameters obtained from the Rietveld refinement. These observations support the coexistence of closely associated multiphase nanodomains within an individual NaLNM particle.

**Figure S3.** The GITT potential-relaxation profiles of (a) NaNM and (b) NaLNM. A current pulse of 0.1 C was applied for 15 min, followed by a 3 h open-circuit relaxation. ΔEτ denotes the voltage change during the current pulse after correction for the instantaneous IR drop, and ΔEs denotes the change between two consecutive relaxed voltages. The voltage drift during the final hour of relaxation was within approximately ±0.2 mV.

**Figure S4** Schematic representation of the NaMO_2_ unit cell. The yellow, gray and red spheres represent Na, M and O atoms, respectively. The atomic displacements from E_g_ and A_1g_ vibrational modes are shown in red and purple arrows, respectively.

**Figure S5** Mn K-edge TFY XANES spectra for (a)NaNM and (b)NaLNM under different charge/discharge states.

**Figure S6** O K-edge FY sXAS data of the pristine electrode and electrodes charged to 4.3V from (a) Na/NaNM and (b) Na/NaLNM.

**Figure S7** (a)Total negative ion, ^16^O^2-^ and ^18^O^2-^ distribution graphs from time-of- flight secondary ion mass spectrometry (TOF-SIMS) images; (b)comparison of selected ion (^16^O^2-^ and ^18^O^2-^) intensity collected from ^16^O^2-^ and ^18^O^2-^ distribution graphs of the ^18^O_2_ labelled pristine NaNM.

**Figure S8** Amount of CO_2_ evolution according to the OEMS results of the ^18^O_2_ labelled NaNM and NaLNM.

**Figure S9** Cross-sectional SEM images of (a) pristine NaNM, (b–d) NaNM after 100 cycles at 1 C, (e) pristine NaLNM, and (f–h) NaLNM after 100 cycles at 1 C. The insets in panels (b) and (f) show enlarged views of representative particle regions.

**Figure S10** Raman spectra of a single particle from pristine electrode and electrodes from cells after 100 cycles of (a) NaNM and (b) NaLNM.
